# Cardiac screening in the armed forces: bridging policy and practice through expert consensus

**DOI:** 10.3389/fcvm.2026.1976017

**Published:** 2026-10-14

**Authors:** Mark Abela

**Affiliations:** 1 Department of Cardiology, Mater Dei Hospital, Msida, Malta; 2 Faculty of Medicine and Surgery, University of Malta, Msida, Malta; 3 Cardiovascular and Genomics Research Institute at St George's, University of London, London, United Kingdom; 4 Medical Centre, Armed Forces of Malta Headquarters, Luqa, Malta

**Keywords:** athletes, cardiac screening, ECG, military, recruitment, sudden cardiac death

## Introduction

Sudden cardiac death in young individuals, though uncommon, remains one of the most devastating events in preventive cardiology. Its occurrence in ostensibly healthy, asymptomatic individuals underscores the limitations of symptom-based risk detection and the need for systematic structured cardiovascular evaluation. This challenge has been recognised across athletic populations, with most sporting bodies and international cardiology societies mandating electrocardiogram (ECG) based cardiac screening in athletes (1–4).

Sudden cardiac death in military personnel is an equally uncommon but deeply consequential event. Thie incidence of sudden cardiac death appears higher when compared to athletic populations, reported as 6.7-11 per 1,000,000 person-years during recruitment and years of service (5–8). This reality is even more pertinent in high-hazard occupations, where individuals are routinely exposed to environments and operational demands that amplify cardiovascular strain. This is especially relevant when immediate medical support is not always guaranteed. These environments can unmask latent cardiovascular disease, accelerate physiological stress responses, and convert otherwise survivable arrhythmic events into fatal outcomes (9). The combination of youth, apparent fitness, and high-risk operational exposure creates a paradoxical vulnerability. Despite these operational concerns, cardiac screening in military recruits is not widespread practice.(10)

The European Association of Preventive Cardiology (EAPC) have recently launched a novel, tiered scalable approach to cardiovascular screening in the Armed Forces (11). These recommendations integrate contemporary scientific evidence with the distinctive realities of military service, balancing operational readiness, logistical constraints, and the early identification of at-risk individuals, while also supporting the physical and mental well being of service members.

## Sudden cardiac death in military recruits

Sudden death in military personnel is estimated to vary between 6.7-11 per 100,000 person-years. The event rate during recruitment training is equally high, with events often taking place during exercise. Despite these concerning numbers, ECG-based cardiac screening is not widespread practice. In a recent international survey, only 71.4% of countries reported including a resting 12-lead ECG at enrolment (10). Despite the high reported incidence of sudden cardiac death in new recruits, the diagnostic yield of cardiac screening program in military populations remains poorly defined (12–15). Studies describing screening outcomes often employ heterogenous methodologies, retrospective designs, ill-defined denominators, and non-standardized screening protocols.

## Mitigating the risk of sudden cardiac death

The health of the force is foundational to mission readiness. Cardiac screening protects not only the individual soldier, but the mission, the unit and the operational integrity of the force. Despite this reality, cardiovascular screening across global Armed Forces remains fragmented, shaped by legacy practices rather than contemporary evidence (10). Cardiac screening is increasingly regarded as a core responsibility rather than a discretionary practice.

The proposed screening algorithm for high level athletes have been endorsed by several international societies for several years (16–20). This is the first attempt by a medical society to propose a uniform cardiac screening protocol for military personnel. The EAPC endorsed proposed structure is both progressive and pragmatic, whilst also acknowledging that not all Military branches have equal access to advanced diagnostics or the relevant cardiology expertise. Such an approach avoids the false dichotomy between no screening and a full cardiac screen. We instead propose a scalable roadmap that can evolve as awareness and resources take shape (11).

## Proposed screening model

In this tiered EAPC endorsed screening model, we outline the essential components in a screening program for military personnel. The consensus statement encourages cardiac screening in all new military recruits, thereby acknowledging the seemingly higher risk of SCD during recruitment training (5–8). The essential triad of a health questionnaire, physical examination, and resting 12-lead ECG reflects the current screening recommendations for athletic populations (Figure 1). The manuscript highlights that a resting ECG is markedly more sensitive than the history or physical examination alone, and that contemporary interpretation criteria have dramatically reduced false positives (11, 21). Extending this to military personnel is not only logical but overdue. This also reflects recent manuscripts supporting ECG based screening models in non-athletic populations (22, 23).

**Figure 1:**
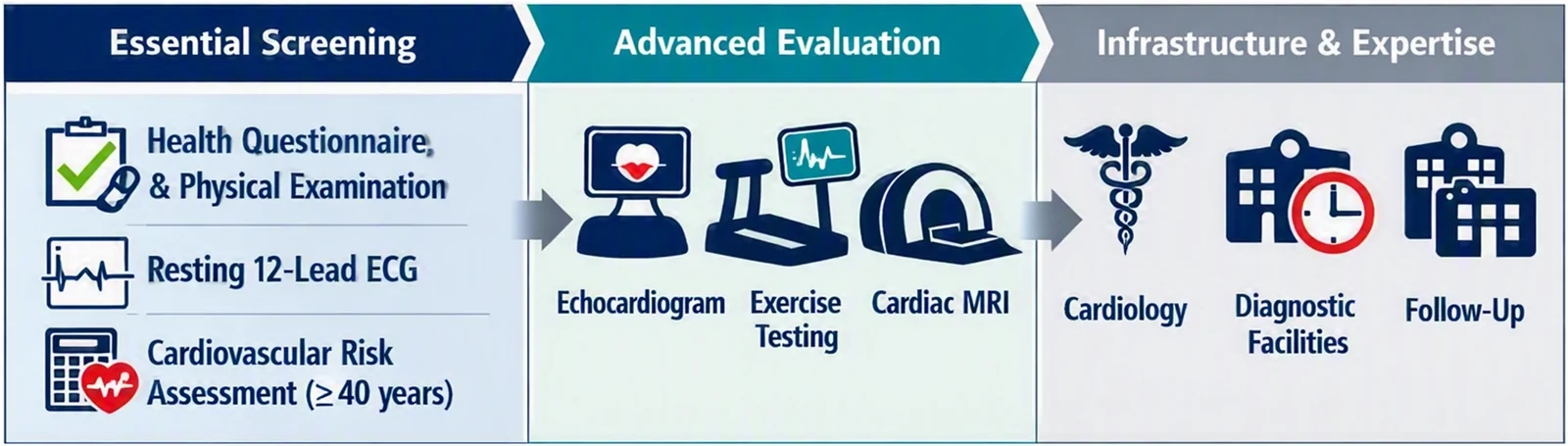
Tiered cardiac screening in military personnel.

The statement also addresses cardiac screening in older individuals, defined as >40 years of age. The essential triad proposed for younger individuals carries a poor sensitivity to detect atherosclerotic cardiovascular disease. Coronary artery disease is the leading cause of sudden cardiac death in older individuals, hence why adapting the screening algorithm is essential (24, 25). We also propose incorporating a cardiovascular risk assessment with validated risk scores (26, 27), with ischaemia testing (functional) and/or coronary anatomy testing (coronary anatomy calcium scoring [CACS] or computed tomography coronary angiography [CTCA]) in those with a high or very high cardiovascular risk. This pragmatic approach highlights at risk asymptomatic individuals, whilst safeguarding resources and cost. We do however acknowledge that the optimal method of determining cardiovascular risk in this healthy asymptomatic population remains unclear, especially with emerging evidence of the high prevalence of coronary calcification and high-risk plaque morphology (not flow limiting) in asymptomatic master athletes and military personnel when screened with CACS/CTCA (28–34).

## Approaching an abnormal screen

As outlined in the statement, referral for second line investigations is paramount in those with symptoms, a family history of inherited cardiovascular disorders and/or sudden cardiac death, signs during physical examination and/or ECG abnormalities (11). Targeted investigations such as echocardiography, exercise testing, cardiac magnetic resonance imaging, ambulatory ECG monitoring, CACS/CTCA, are guided by symptoms, abnormal findings, or elevated risk. Infrastructure and expertise play a key role in personalizing the evaluation. Individuals with a diagnosis are then counselled within a multidisciplinary team framework. We also encourage periodic re-evaluation in individuals with early phenotypic traits of cardiovascular disease (11).

## Balancing autonomy and risk: does the shared decision model apply?

The shared decision-making model has reshaped exercise recommendations in athletes with cardiovascular disease. Athlete autonomy plays a key role in the process, moving a way from the traditional paternalistic approach (3, 4).

Do these concepts apply for military personnel? Cardiovascular readiness has become a defining component of military capability. These individuals are often expected to perform in austere, high stress, and medically remote environments. Individuals with cardiovascular disease exposed to these conditions may inevitably experience symptoms or clinically adverse events. A single event can compromise mission execution, jeopardise colleagues, and erode confidence within tightly bonded units. The consensus statement acknowledges that the shared decision-making model may not be suitable in this unique population, highlighting that a command directed approach is often required to safeguard the individual's safety and the institution's objectives (11). A recent tactical athletes consensus statement also echoes this proposed shift in practice (35). The tactical clinical management (TCM) model is a deliberative risk assessment process that factors in the individual's preferences with the realities of operational safety, incorporating task-specific capability, environmental stressors, team and public-safety implications.

## Infrastructure and expertise

This novel, tiered screening framework attempts to bring coherence to this military landscape. Its strength lies not only in the scientific rationale but in its pragmatic design. The authors recognize that military organisations differ widely in resources, expertise, and infrastructure (11). Screening is only as strong as the system that supports it, infrastructure readiness is a prerequisite for implementation (Figure 2). This sentiment is also acknowledged in the recent ACC/AHA scientific statement on Tactical Athletes (35), which similarly emphasize that operationally informed risk-assessment should be made in the right environment, lead by experts and backed with the right infrastructure. Having i) access to the right clinicians, ii) clear referral pathways, iii) timely access to second line investigations, iv) mechanisms for longitudinal follow up, and v) data systems designed to monitor screening outcomes and clinical events help ensure excellence. These aspects help address knowledge gaps, refine protocols and support quality assurance initiatives.

**Figure 2:**
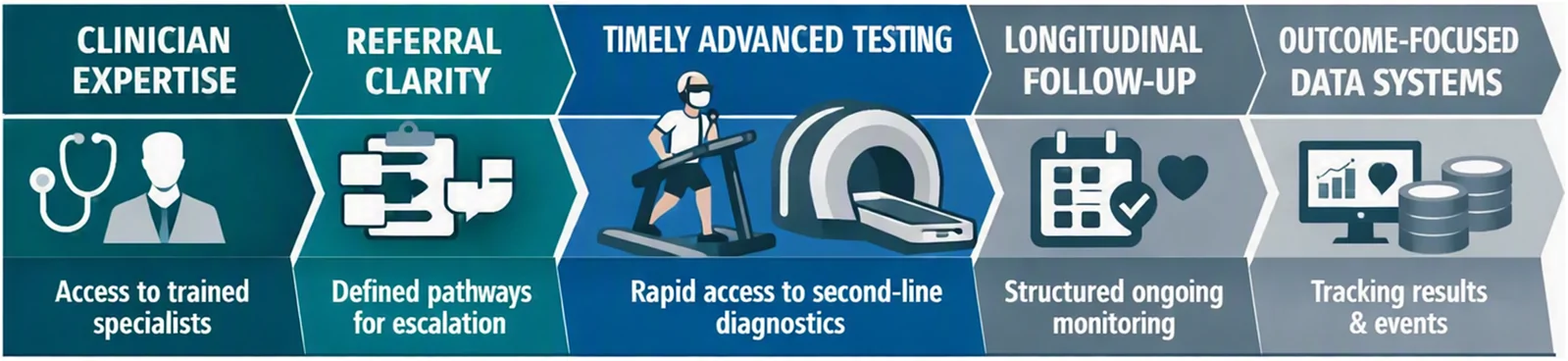
Infrastructure readiness for cardiac screening.

## Conclusions

Military forces have a unique responsibility, to safeguard the healthy of individuals who operate in environments where medical failure carries disproportionate consequences. A tiered, infrastructure-dependent cardiovascular screening framework offers a modern, evidence-aligned solution. The consensus statement is novel in its structure, pragmatic in its application, and adaptable across branches and nations. Cardiovascular screening is a strategic necessity that protects is service members whilst strengthens operational capability, aligning military practice with the best available evidence. This is the moment for military organisations to move beyond fragmented legacy approaches, instead adopting a unified, scalable model that ensures service personnel with cardiovascular disease are identified early in their career, managed appropriately, and never left to chance.
